# Supplementary material for: A novel type of colistin resistance genes selected from random sequence space
Source: PLoS Genet. 2021 Jan 7;17(1):e1009227. doi: 10.1371/journal.pgen.1009227 (PMC7790251; doi:10.1371/journal.pgen.1009227)
Supplement: S1 Table — Only one site aligns as a group with strongly similar properties (indicated with ‘:’). No residues group with perfect (‘*’) or weak (‘.’) similarities. (DOCX) [file pgen.1009227.s001.docx]

**S1 Table. Multiple sequence alignment of Dcr1-6 using Clustal Omega.** Only one site aligns as a group with strongly similar properties (indicated with ‘:’). No residues group with perfect (‘*’) or weak (‘.’) similarities.

Dcr1 -MSLFVSITFLMCII-----FLCILIMT------ITL-TL-----------LSL-------- 30

Dcr2 ---------MLMFALVPLSLIFIISLLLITLVLSLPVL--PVLVILTLTMLLSAMVVVILPN 51

Dcr3 ---------------MPIIWFITITVLL----ASLLLVIFLVFVVLLV---IRFTFILI--- 37

Dcr4 ---MIILVMTIIIIIVPLLTLFV----ILTMLSSSIVI--PVFVLLTL--FLALFVIAFVTN 51

Dcr5 MVTTLIVLVYLIAIVLAFSFFMPTLLMT------SPMLTLSTLVV-----LLALLLLV-FSN 50

Dcr6 ---------------------MIVAIII---VHSLNHI--M-LMLLLLSLLYR--------- 26

:
