# Supplementary material for: A novel type of colistin resistance genes selected from random sequence space
Source: PLoS Genet. 2021 Jan 7;17(1):e1009227. doi: 10.1371/journal.pgen.1009227 (PMC7790251; doi:10.1371/journal.pgen.1009227)
Supplement: S2 Table — MICs of tetracycline (TET), ciprofloxacin (CIP), streptomycin (STR), chloramphenicol (CHL), ertapenem (ETP), erythromycin (ERY) and ceftazidime (CAZ) were determined using Etest on agar plates. ND = not determined. MICs for colistin were determined using Sensititre plates (Thermo fisher). All MIC determinations have been performed at least in triplicates. (DOCX) [file pgen.1009227.s002.docx]

**S2 Table. Minimal inhibitory concentrations (MIC) of selected inserts in various backgrounds.** MICs of tetracycline (TET), ciprofloxacin (CIP), streptomycin (STR), chloramphenicol (CHL), ertapenem (ETP), erythromycin (ERY) and ceftazidime (CAZ) were determined using Etest on agar plates. ND = not determined. MICs for colistin were determined using Sensititre plates (Thermo fisher). All MIC determinations have been performed at least in triplicates.

| Strain | MIC (mg/L) | | | | | | | |
| --- | --- | --- | --- | --- | --- | --- | --- | --- |
|  | CST | TET | CIP | STR | CHL | ETP | ERY | CAZ |
| *E. coli* BW25113 /pRD2(empty) | 0.5 | 1.5 | 0.008 | 2 | 3 | 0.002 | 16 | 0.19 |
| *E. coli* BW25113 /pRD2(*dcr1*) | 8 | 1 | 0.008 | 2 | 3 | 0.004 | 16 | 0.19 |
| *E. coli* BW25113 /pRD2(*dcr2*) | 8 | 1.5 | 0.008 | 2 | 3 | 0.004 | 24 | 0.38 |
| *E. coli* BW25113 /pRD2(*dcr3*) | 4 | ND | ND | ND | ND | ND | ND | ND |
| *E. coli* BW25113 /pRD2(*dcr4*) | 4 | ND | ND | ND | ND | ND | ND | ND |
| *E. coli* BW25113 /pRD2(*dcr5*) | 4 | ND | ND | ND | ND | ND | ND | ND |
| *E. coli* BW25113 /pRD2(*dcr6*) | 4 | ND | ND | ND | ND | ND | ND | ND |
| *E. coli* MG1655 /pBAD18(empty) | 0.5 | ND | ND | ND | ND | ND | ND | ND |
| *E. coli* MG1655 /pBAD18(*dcr1*) | 8 | ND | ND | ND | ND | ND | ND | ND |
| *E. coli* MG1655 /pBAD18(*dcr2*) | 4 | ND | ND | ND | ND | ND | ND | ND |
| *S.* Typhimurium LT2 /pBAD18(empty) | 1 | ND | ND | ND | ND | ND | ND | ND |
| *S.* Typhimurium LT2 /pBAD18(*dcr1*) | 8 | ND | ND | ND | ND | ND | ND | ND |
| *S.* Typhimurium LT2 /pBAD18(*dcr2*) | 8 | ND | ND | ND | ND | ND | ND | ND |
| *K. pneumoniae* ATCC13883 /pRD2(empty) | 2 | ND | ND | ND | ND | ND | ND | ND |
| *K. pneumoniae* ATCC13883 /pRD2(*dcr1*) | 8 | ND | ND | ND | ND | ND | ND | ND |
| *K. pneumoniae* ATCC13883 /pRD2(*dcr2*) | 8 | ND | ND | ND | ND | ND | ND | ND |
