# Supplementary material for: A novel type of colistin resistance genes selected from random sequence space
Source: PLoS Genet. 2021 Jan 7;17(1):e1009227. doi: 10.1371/journal.pgen.1009227 (PMC7790251; doi:10.1371/journal.pgen.1009227)
Supplement: S3 Table — Shown are the mean of four biological and two technical replicates each. Numbers in parentheses represent the standard deviation. (DOCX) [file pgen.1009227.s003.docx]

**S3 Table.** Relative growth rates of *dcr1*-expressing strains compared to a wild-type control strain. Shown are the mean of four biological and two technical replicates each. Numbers in parentheses represent the standard deviation.

| Strain | Relative growth rate (SD) |
| --- | --- |
| *E. coli* BW25113 wild type | 1.00 (0.02) |
| *E. coli* BW25113 ∆*bglGFB*::PLlacO-*dcr1* | 0.98 (0.01) |
| *E. coli* BW25113 ∆*bglGFB*::PLlacO -*dcr2* | 1.01 (0.01) |
| *E. coli* BW25113 ∆*bglGFB*::PLlacO -*dcr3* | 1.01 (0.01) |
| *E. coli* BW25113 ∆*bglGFB*::PLlacO -*dcr4* | 1.01 (0.01) |
| *E. coli* BW25113 ∆*bglGFB*::PLlacO -*dcr5* | 1.03 (0.01) |
| *E. coli* BW25113 ∆*bglGFB*::PLlacO -*dcr6* | 1.00 (0.02) |
| *E. coli* BW25113 ∆*bglGFB*::J23101 –*dcr1* | 1.02 (0.01) |
