# Supplementary material for: A novel type of colistin resistance genes selected from random sequence space
Source: PLoS Genet. 2021 Jan 7;17(1):e1009227. doi: 10.1371/journal.pgen.1009227 (PMC7790251; doi:10.1371/journal.pgen.1009227)
Supplement: S1 Fig — Changes in the nucleotide and amino acid sequence are shown in orange. (DOCX) [file pgen.1009227.s004.docx]

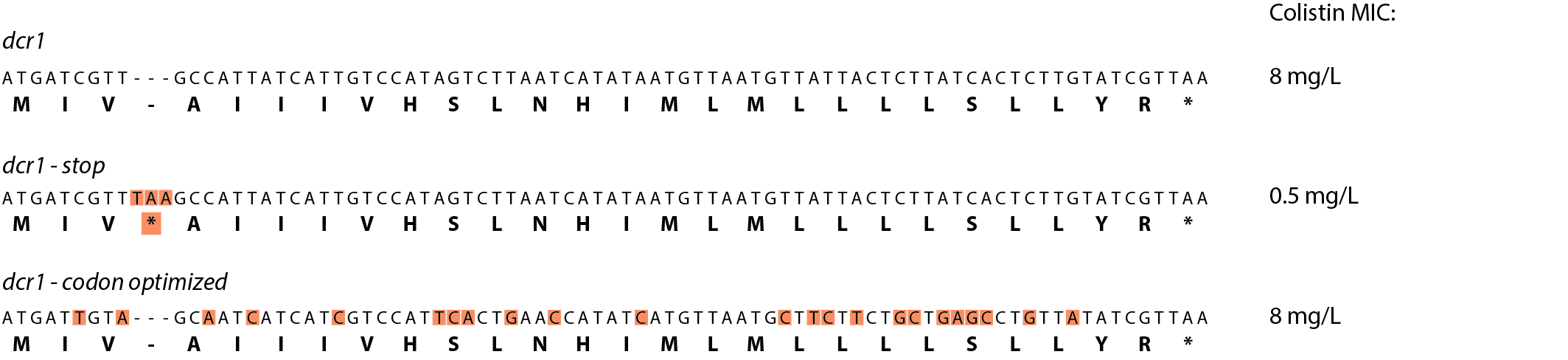


**S1 Fig. MICs of colistin for Dcr1 peptide variants.** Changes in the nucleotide and amino acid sequence are shown in orange.
