# Supplementary material for: A novel type of colistin resistance genes selected from random sequence space
Source: PLoS Genet. 2021 Jan 7;17(1):e1009227. doi: 10.1371/journal.pgen.1009227 (PMC7790251; doi:10.1371/journal.pgen.1009227)
Supplement: S2 Fig — Expression of Dcr peptides does not cause severe defects in lag time or final yield when expressed from the plasmid pRD2 (A) or from a single chromosomal copy under various promoters (B). The points represent the mean of at least three biological and two technical replicates and error bars show the standard deviation. All measurements were performed in presence of 1mM IPTG (inducer). (DOCX) [file pgen.1009227.s005.docx]

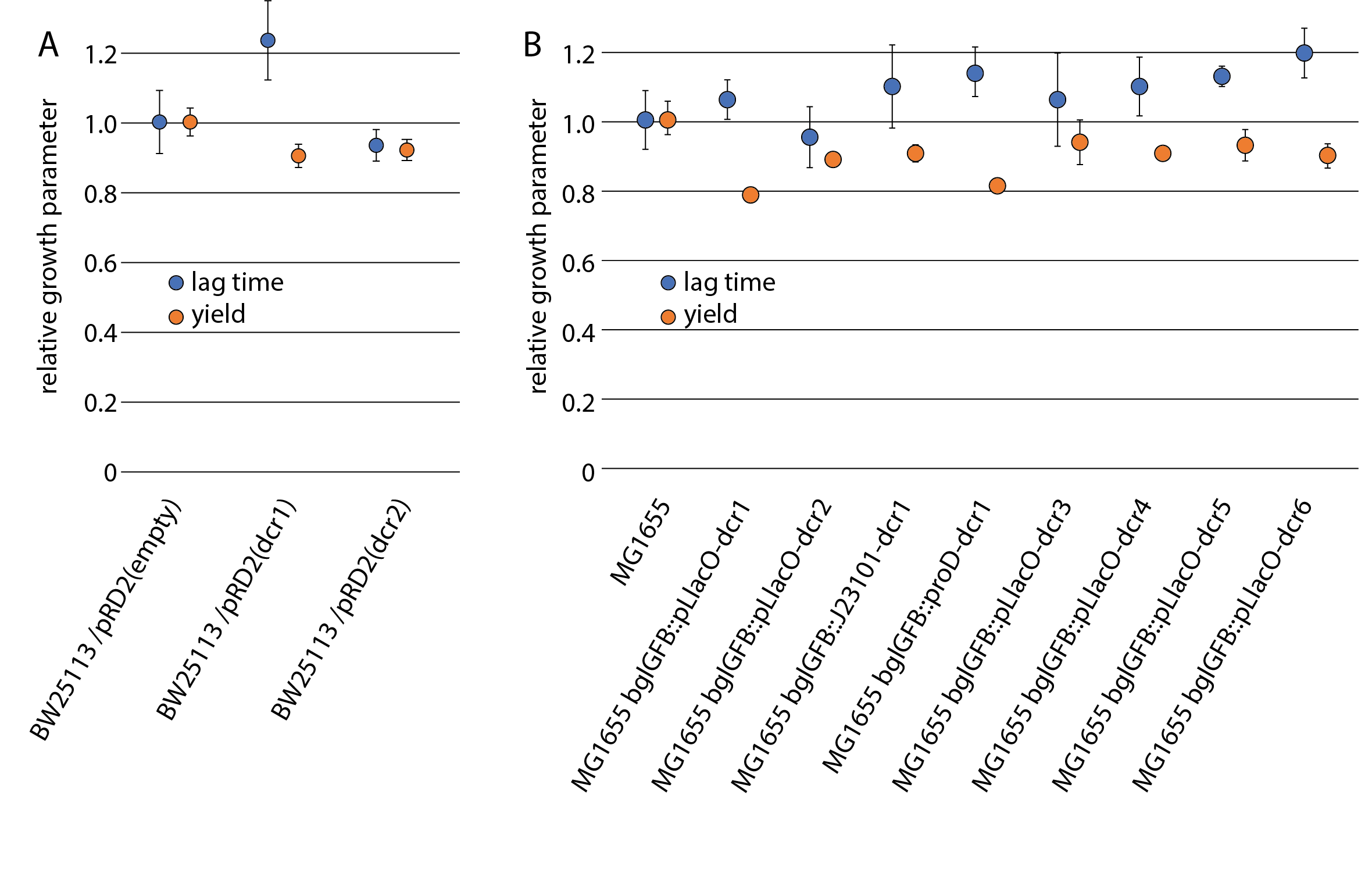


**S2 Fig. Growth characteristics of Dcr peptides**. Expression of Dcr peptides does not cause severe defects in lag time or final yield when expressed from the plasmid pRD2 (A) or from a single chromosomal copy under various promoters (B). The points represent the mean of at least three biological and two technical replicates and error bars show the standard deviation. All measurements were performed in presence of 1mM IPTG (inducer).
