# Supplementary material for: A novel type of colistin resistance genes selected from random sequence space
Source: PLoS Genet. 2021 Jan 7;17(1):e1009227. doi: 10.1371/journal.pgen.1009227 (PMC7790251; doi:10.1371/journal.pgen.1009227)
Supplement: S3 Fig — MICs for colistin were determined using Sensititre plates (Thermo fisher). All MIC determinations have been performed at least in triplicates. (DOCX) [file pgen.1009227.s006.docx]

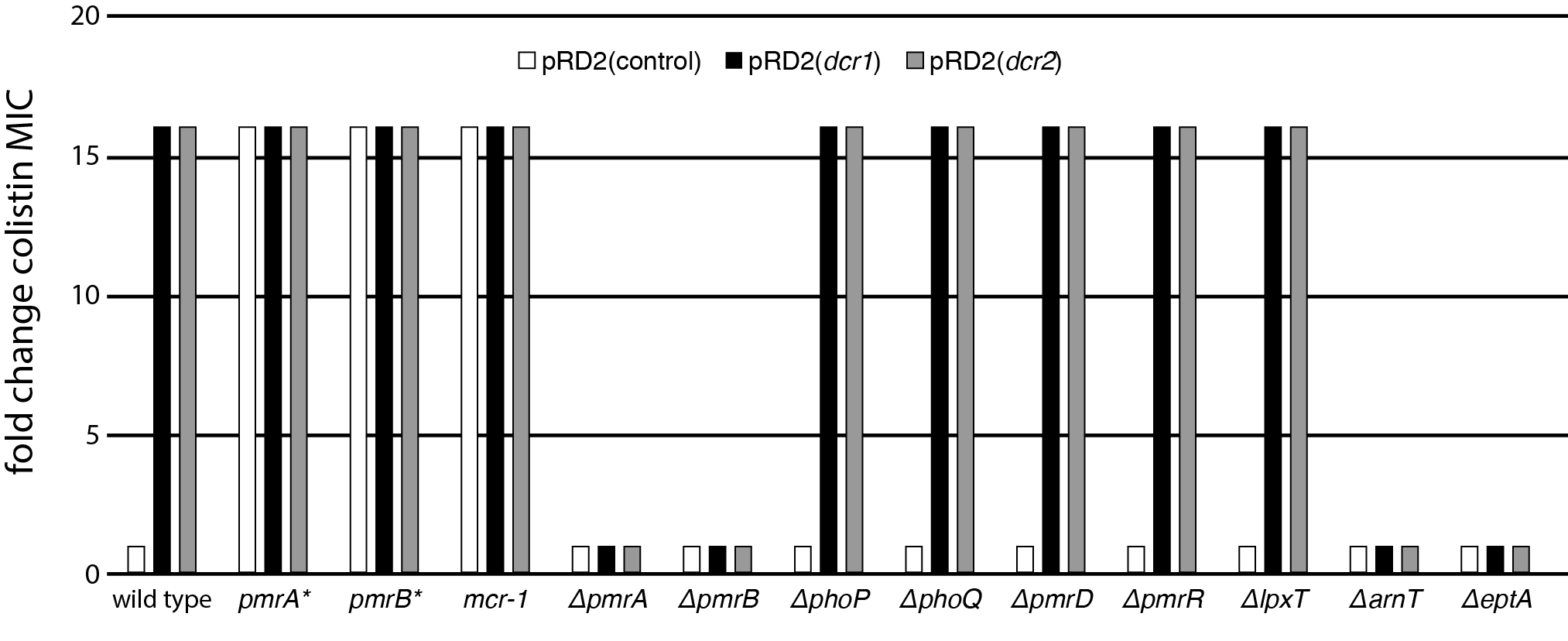


**S3 Fig. MICs of colistin for plasmid-encoded Dcr1 and Dcr2 and the empty control vector in various genetic backgrounds of *E. coli* BW25113.** MICs for colistin were determined using Sensititre plates (Thermo fisher). All MIC determinations have been performed at least in triplicates.
