# Supplementary material for: A novel type of colistin resistance genes selected from random sequence space
Source: PLoS Genet. 2021 Jan 7;17(1):e1009227. doi: 10.1371/journal.pgen.1009227 (PMC7790251; doi:10.1371/journal.pgen.1009227)
Supplement: Attachment — Submitted filename: Response letter final.docx [file pgen.1009227.s007.docx]

Reviewer #1: This is an interesting paper in that it predicts a mechanism by which bacteria could evolve resistance to antimicrobials via activation of TCSs. I found the approach to be novel and accept the reported findings. I do have a few points for the authors to consider:
1. Have the authors prepared synthetic peptides corresponding to those identifed and ascertained whether they bind to PmrB in vitro? If such peptides can be made soluble, which may be an issue, would their addition to intact bacteria result in activation of PmrB?

This is a good point, which we also have considered. However, as the reviewer suspected, in vitro experiments are greatly limited by the high hydrophobicity of the peptides causing very limited solubility. We therefore focused on showing *in vivo* interactions under physiological conditions which we believe are sufficient to show a direct interaction between PmrB and the novel peptides.

Regarding addition of peptide to intact bacteria this is very unlikely to work since such long peptides (26 to 51 amino acids) are not taken up by bacteria without first being degraded to shorter fragments (S. enterica and E. coli typically can transport oligopeptides consisting of 3 to 4 amino acids via the OppA system).

2. In theory, the approach could be used to sensitize bacteria to cationic antimicrobial peptides via inhibiting PmrB. Perhaps the authors could discuss this possibility to assist in devloping adjunctive therapies?

The reviewer raises a very interesting point. If PmrB-activators can be selected, it is conceivable that inhibitors can be selected in a similar way We have added this consideration to our discussion (lines 268-270).

3. I note that some of the experimental results, particularly MIC values, are from only duplicate experiments. For the sake of rigor, results from triplicate experiments should be reported as mode values.

We agree and while many MICs were already based on triplicates, we have now replicated all reported MIC’s at least three times.

Reviewer #2: The authors describe their work to identify and characterise novel peptides that, when expressed in E. coli, lead to resistance to the antibiotic colistin. The work is performed to a high standard and the conclusions are supported by the data. The experiments to determine that one of the identified ‘synthetic’ colistin resistance peptides acts through directly interacting with the two-component sensor histidine kinase BasS/PmrB are particularly elegant.

Throughout the manuscript the authors discuss the two-component system PmrAB, but this system is termed BasRS in E. coli (Nagasawa et al. J Biochem. 1993 114(3):350-7.) while the name PmrAB is used in other Gram-negatives (Salmonella, Klebsiella). I would recommend the authors to use the term BasRS throughout the manuscript, or, at a minimum, indicate that BasRS is a synonym for this two-component system in E. coli. Indeed, I notice that the authors use BasS in Fig. 2D.

We thank the reviewer for pointing this out. We have kept the PmrAB name but indicated at two places (Abstract and Introduction, lines 32, 88) BasRS is the proper terminology for E. coli.

In addition, there are data lacking in the manuscript (and these need to be added), and some typos that need fixing. These are further outlined below.

L. 55: correct ‘pear’ to ‘per’

Corrected.

L. 100: within the context of this study, it is not entirely obvious why the authors have chosen to make a library based on amino acids that have been present on the primordial Earth. This should be explained in more detail. Perhaps more importantly, I was unable to find information on the libraries from which Dcr1-6 were selected and this information should be added to the manuscript.

This is a good point. We have changed the phrasing to better clarify the reason for choosing a primordial library (lines 101-102). We also clarified from which libraries the peptides were isolated (lines 114-116).

L. 116 - 120: for clarity, the rationale for generating the chromosomal construct may be described first, before the results are discussed.

We have clarified this (lines 122-123).

L. 130: the data for Klebsiella and Salmonella are currently not included in the manuscript but should be provided.

We have added the absolute MIC values for Kpn and Sal to Supplementary Table 2.

L. 159 - 178: this section is confusing as it discusses Dcr-3, but the data in Fig. 3 appear to show data for another Dcr (Dcr-1). This should be checked and corrected. Similar to my comment on Fig 3 (see below), the heading of this section should be changed as data for only one Dcr are provided. Indeed, the data discussed in L.168-169 need to be provided in the manuscript.

We apologize for this error. We have indeed mislabeled the figure and we have corrected it.

The loss of activity for the DCR1/2/3 fusions was not quantified but clearly visible on McConkey agar. Additionally, the fusions no longer provided resistance, showing that they lost functionality. After we were able to select a functional linker for Dcr3 as described in the manuscript, we also included the original fusion (without linker) of Dcr3 in the beta-gal assay as seen in Figure 3c (pUT18c-PmrB with pKT25-Dcr3, showing no activity above background). We have clarified this in the figure legend.

L. 192: please add the heading ‘Discussion’ here.

We have added the headings ‘Introduction’, ‘Results’ and ‘Discussion.’

L. 297: correct ‘over night’ to ‘overnight’

Corrected.

L. 298: complete line after 100 mg/L.

Completed.

L. 314: ‘appropriate antibiotic’ is perhaps somewhat ambiguous. Can the authors be more specific and confirm that they did not include colistin in the plates on which they selected transformants?

We thank the reviewer for pointing this out. Indeed, it is important to clarify that colistin was not included for selection of the re-transformants. We have specified which antibiotics were used for the plasmid transformations.

Fig. 3. The title of the figure should be changed to reflect that this figure does not show data of multiple peptides identified in this study, but only the Dcr-1 peptides (see also comment above). In panel (B), correct ‘vaiants’ to ‘variants’

This has been corrected.

Reviewer #3: This is an interesting manuscript describing the results of an experiment where over 100 million randomly generated DNA sequences in Escherichia coli were tested for resistance to colistin. This resulted in six variants that encoded peptides providing resistance. It is shown that these peptides are activators of a two-component system resulting in decreased antibiotic uptake. This is interesting for the topic of antibiotic resistance (colistin being a last resort antibiotic) and for the topic of evolution of novel functions (since the function emerges from random sequences). This goes in the line of previous ideas that non-coding DNA can serve as a substrate for de novo gene evolution. The findings are very interesting and worthy of publication. The paper is clear and well-written.

I first gave some consideration on whether this is novel enough for PlosG. The approach was published before by the authors in a mBIO paper focusing on aminoglycosides where it was also found that small peptides affect membrane structure and thus antibiotic uptake. I do think the present manuscript is novel enough because it focuses on another antibiotic, the peptides are not the same and the discussion around the evolutionary consequences of the results is much more developed here than in the previous publication.

The methods detailing the computational analyses are not precise enough. One needs proper citations of the methods, indication of version numbers, and of the parameters that were used to run the program and filter the significant results (where relevant).

We apologize for the lack of detail for some of the analyses. We have clarified this (lines 101-102), as well as in Materials and Methods and added Supplementary Table 1.

Line 112. I would have expected the presentation of some evidence for no cross resistance with other antibiotics. It's an important point.

We apologize for the lack of clarity. We are showing the cross-resistance analysis in Figure 1d, but did not indicate it clearly in the text. We have added this in the text (lines 117-118) and also added the absolute values to Supplementary Table 2.

The discussion on small random genes being possible in genomes is interesting. It might merit some complement, since recombination/fusion can incorporate these genes into larger ones and thus provide novel functional domains to existing proteins.

This is an excellent point. We have added this to our Discussion (lines 244-246).

The manuscript lacks a clear separation into introduction, results, discussion, etc. To the best of my knowledge this does not fit the format of PlosG.

We have changed to the correct format.
